# Combined Effects of Combined Resistance Exercise and Vitamin D Supplementation on Cardiometabolic Profiles and Functional Capacities in Elderly Women with Type 2 Diabetes

**DOI:** 10.3390/metabo16080593

**Published:** 2026-08-19

**Authors:** Hyoung-Jun Kim, Deok-Su Yoo, Man-Gyoon Lee

**Affiliations:** 1 Graduate School of Physical Education, Kyung Hee University, 1732 Deogyeong-daero, Giheung-gu, Yongin-si 17104, Gyeonggi-do, Republic of Korea; giant0486@gmail.com; 2 Department of Taekwondo, College of Physical Education, Kyung Hee University, 1732 Deogyeong-daero, Giheung-gu, Yongin-si 17104, Gyeonggi-do, Republic of Korea

**Keywords:** type 2 diabetes mellitus, resistance training, vitamin D supplementation, elderly women, cardiometabolic profiles, functional capacities, glycemic control, calcitonin, postural balance

## Abstract

**Background/Objectives**: This 12-week randomized controlled trial investigated whether progressive resistance training combined with vitamin D supplementation produces enhanced combined improvements in cardiometabolic profiles and functional capacities compared with monotherapies in elderly Korean women with type 2 diabetes mellitus (T2DM) and vitamin D deficiency. **Methods**: In a 2 × 2 factorial design, 52 women (aged 65–80 years) with T2DM and serum 25(OH)D < 20 ng/mL were assigned to four groups: Exercise + Vitamin D (Ex + VitD, *n* = 15), Exercise + Placebo (Ex + Placebo, *n* = 13), Vitamin D only (VitD, *n* = 11), or Control (*n* = 13). Exercise groups completed supervised progressive resistance training three times weekly, and vitamin D groups received 2000 IU/day cholecalciferol. **Results**: The Ex + VitD group achieved significant improvements in HbA1c (−0.13%, *p* = 0.019), fasting glucose (−0.79 mmol/L, *p* < 0.001), insulin (−1.96 μU/mL, *p* = 0.050), and HOMA-IR (−0.77, *p* = 0.020). Serum 25(OH)D increased substantially (+15.57 ng/mL, *p* < 0.001). Calcitonin rose exclusively in the Ex + VitD group (+2.57 pg/mL, *p* < 0.001, d = 3.34), while monotherapies showed no change. Total cholesterol (−23.93 mg/dL), triglycerides (−25.07 mg/dL), and LDL-cholesterol (−10.20 mg/dL) decreased significantly. Both exercise groups showed marked strength gains (chair stand +8.33 to +10.00 repetitions, *p* < 0.001) and balance improvements (functional reach +2.47 to +4.46 cm, *p* ≤ 0.033). **Conclusions**: Twelve weeks of progressive resistance training combined with vitamin D supplementation produced enhanced combined improvements in glycemic control, insulin sensitivity, calcitonin secretion, muscular strength, and lipid metabolism in elderly women with T2DM and vitamin D deficiency, targeting complementary pathways that neither intervention engaged alone.

## 1. Introduction

Among South Koreans aged ≥65 years, type 2 diabetes mellitus (T2DM) affects nearly one-third of the population, producing severe consequences such as a three-fold elevated fall incidence [1] and increased cardiovascular mortality [2]. More than 70% of elderly Korean diabetics exhibit vitamin D deficiency (serum 25(OH)D < 20 ng/mL), a modifiable risk factor that influences insulin secretion and glucose uptake via receptors expressed in β-cells, skeletal myocytes, and adipocytes [3,4]. Clinical trials of vitamin D monotherapy have yielded inconsistent outcomes, with meta-analyses documenting only modest HbA1c reductions (0.2–0.4%) [5,6].

As an evidence-based cornerstone of diabetes management, progressive resistance training activates glucose transporter type 4 (GLUT4) translocation through insulin-independent pathways during muscle contraction [7]. In a 16-week progressive resistance protocol, Castaneda et al. reported clinically meaningful HbA1c reductions (−1.1%), with nearly three-quarters of participants reducing diabetes medication [8]. Resistance training also improves neuromuscular coordination and postural stability, thereby reducing fall risk [9,10].

Recent evidence indicates that combined interventions may yield combined effects. Studies in diabetic rodents show that exercise and vitamin D supplementation cooperatively enhance hepatic peroxisome proliferator-activated receptor gamma (PPARγ) expression beyond levels achieved with monotherapy [4]. Clinical trials by Farag et al. [11,12] and Sun et al. [13] have documented superior metabolic improvements with combined interventions. Nevertheless, research focusing on elderly Asian women with both T2DM and vitamin D deficiency remains limited. Kim et al. reported body composition improvements with circuit training and vitamin D supplementation but found no glycemic effects, possibly owing to the mixed aerobic–resistance modality and the exclusion of bone metabolism markers [14].

Elderly diabetics are vulnerable to falls because of compromised postural stability arising from peripheral neuropathy and proprioceptive deficits [15,16]. Although advanced systems such as Posturomed allow precise biomechanical quantification of dynamic stability [17], no study has examined the effects of combined interventions on postural control using such technology.

Beyond its classical role in calcium and bone homeostasis, emerging evidence highlights calcitonin as an endocrine factor responsive to both mechanical strain and nutritional status. Thyroid C-cells express functional vitamin D receptors and exhibit secretory responsiveness to physical exercise and mechanical stress [18]. Importantly, calcitonin receptor signaling in pancreatic β-cells and metabolic tissues has been implicated in modulating insulin secretion, optimizing cellular energy expenditure, and contributing to systemic cardiometabolic regulation [19]. However, whether combined mechanical stress (resistance training) and vitamin D repletion exert a cooperative action on calcitonin secretion, and whether such alterations mediate cardiometabolic adaptations in type 2 diabetes, remains uninvestigated.

The present randomized trial examined whether 12 weeks of progressive resistance training combined with vitamin D supplementation yields superior improvements in glycemic regulation, vitamin D status, bone metabolism markers, and postural balance compared with monotherapies in elderly Korean women. We hypothesized that the combined intervention would produce superior improvements in glycemic control and functional capacity compared with monotherapies, and explored whether serum calcitonin responses are associated with these combined cardiometabolic adaptations.

## 2. Materials and Methods

### 2.1. Participants

**Figure 1:**
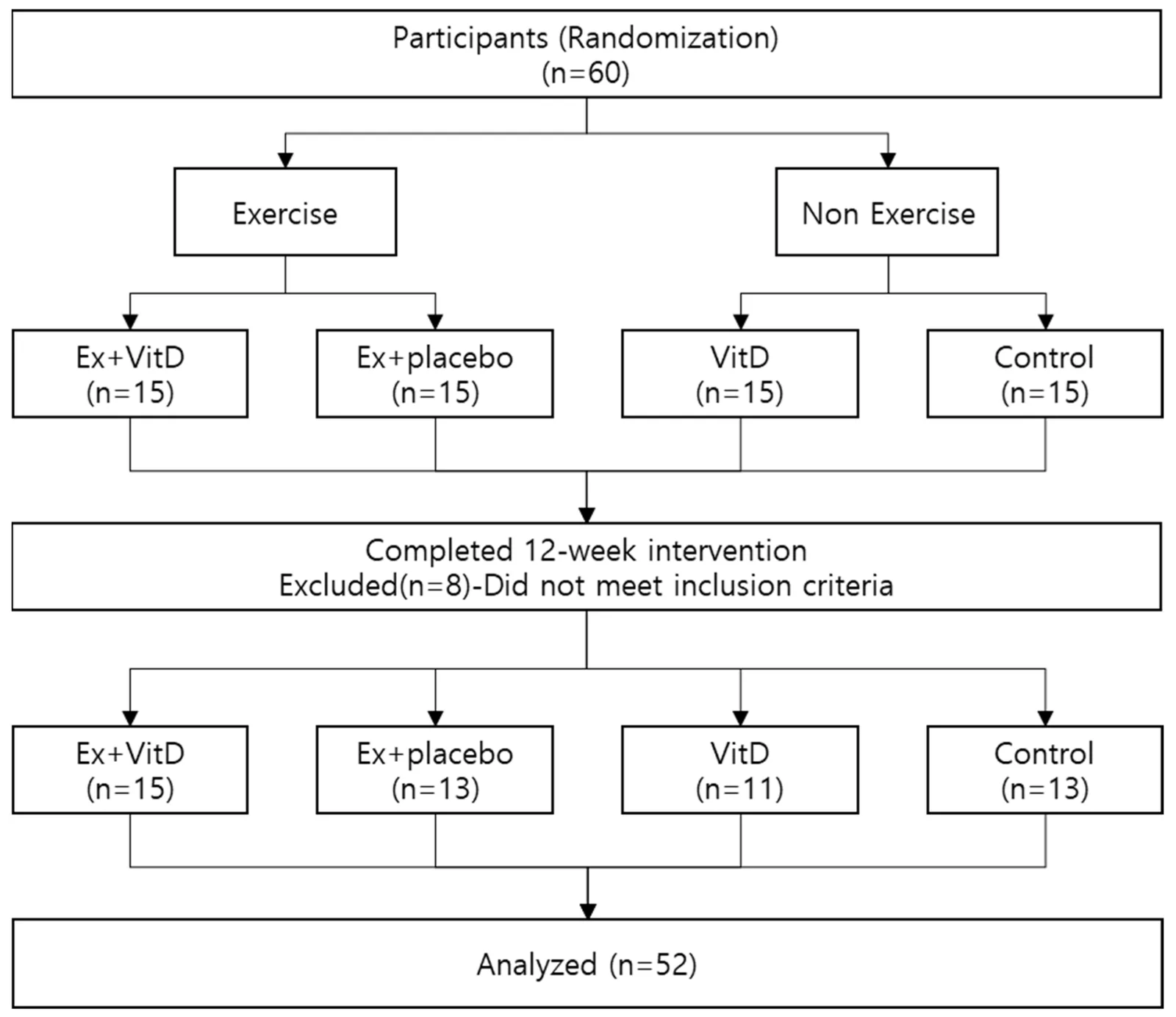
CONSORT flow diagram of participant recruitment, allocation, and retention throughout the 12-week intervention. Of 60 eligible women enrolled and randomized, 8 participants withdrew during the intervention, resulting in 52 participants completing the study across four groups: Exercise + Vitamin D (*n* = 15), Exercise + Placebo (*n* = 13), Vitamin D only (*n* = 11), and Control (*n* = 13).

Community-dwelling women aged 65–80 years with T2DM and vitamin D deficiency were recruited from the Seoul metropolitan area (March–September 2013). To minimize seasonal confounding on serum 25(OH)D levels, participant recruitment and baseline/post-intervention assessments for all four groups were balanced and conducted concurrently within this period. Participants were instructed to maintain their habitual sun exposure and outdoor activity patterns throughout the study. Inclusion criteria were confirmed T2DM (fasting glucose ≥ 126 mg/dL or oral medication), serum 25(OH)D < 20 ng/mL, a sedentary lifestyle (<2 exercise sessions/week for ≥6 months), independent ambulation, and stable medications for ≥3 months. Exclusion criteria were insulin therapy or HbA1c > 10%, medical history of severe cardiovascular disease, uncontrolled hypertension (systolic > 160 mmHg or diastolic > 100 mmHg), severe osteoarthritis, neurological disorders affecting balance/mobility, baseline vitamin D supplementation > 400 IU/day within the preceding 3 months, history of hypercalcemia, and cognitive impairment. An a priori sample size calculation was performed using G*Power (version 3.1.5; Heinrich Heine University Düsseldorf, Düsseldorf, Germany) based on the primary outcome measure, glycated hemoglobin (HbA1c). Assuming a large effect size (f = 0.40) based on prior literature and recruitment feasibility in elderly women with T2DM, a minimum sample size of 52 participants was required for a 2 × 2 factorial ANOVA design (a = 0.05, statistical power 1 − β = 0.80). To account for potential dropouts during the 12-week intervention, a total of 60 eligible participants were enrolled. A total of 60 eligible women were enrolled and randomly assigned to the four groups. During the 12-week trial, 8 participants withdrew due to non-study-related personal reasons, resulting in an overall completion rate of 86.7% (52/60). Ultimately, 52 participants completed the protocol and were included in the final analysis: Ex + VitD (*n* = 15), Ex + Placebo (*n* = 13), VitD (*n* = 11), and Control (*n* = 13). Detailed participant flow is presented in Figure 1.

### 2.2. Study Design and Randomization

This 2 × 2 factorial randomized controlled trial examined the independent and interactive effects of resistance exercise training and vitamin D supplementation over 12 weeks. Participants were randomly assigned to (1) Exercise + Vitamin D (Ex + VitD), (2) Exercise + Placebo (Ex + Placebo), (3) Vitamin D only (VitD), or (4) Control using a computer-generated random block sequence (block size of 4) managed by an independent statistician. The allocation was strictly random and was not targeted or stratified based on baseline physical fitness, clinical status, or demographic variables. Outcome assessments were performed at baseline and week 12. A partial blinding strategy was implemented owing to the nature of the interventions. Double-blinding was maintained for the nutritional intervention: both participants and research staff managing supplement administration were blinded to the vitamin D or placebo allocation using identical-appearing capsules in coded bottles. Due to the inherent nature of exercise training, participants and exercise instructors could not be blinded to exercise group assignment. However, to ensure objectivity and prevent bias, all outcome assessors, laboratory personnel performing biochemical assays, and data analysts remained strictly blinded to participant group allocation throughout the trial and subsequent data analysis.

To minimize confounding variables, participants were required to be on a stable dosage of prescribed anti-diabetic and antihypertensive medications for at least 1 month prior to enrollment and were instructed to maintain their exact regimens throughout the 12-week study period under medical supervision. Furthermore, total daily energy intake was quantitatively tracked using a 3-day dietary log (analyzed via CAN-Pro, version 4.0; The Korean Nutrition Society, Seoul, Republic of Korea), and daily outdoor sunlight exposure (08:00–18:00) was monitored using daily activity logs.

**Figure 2:**
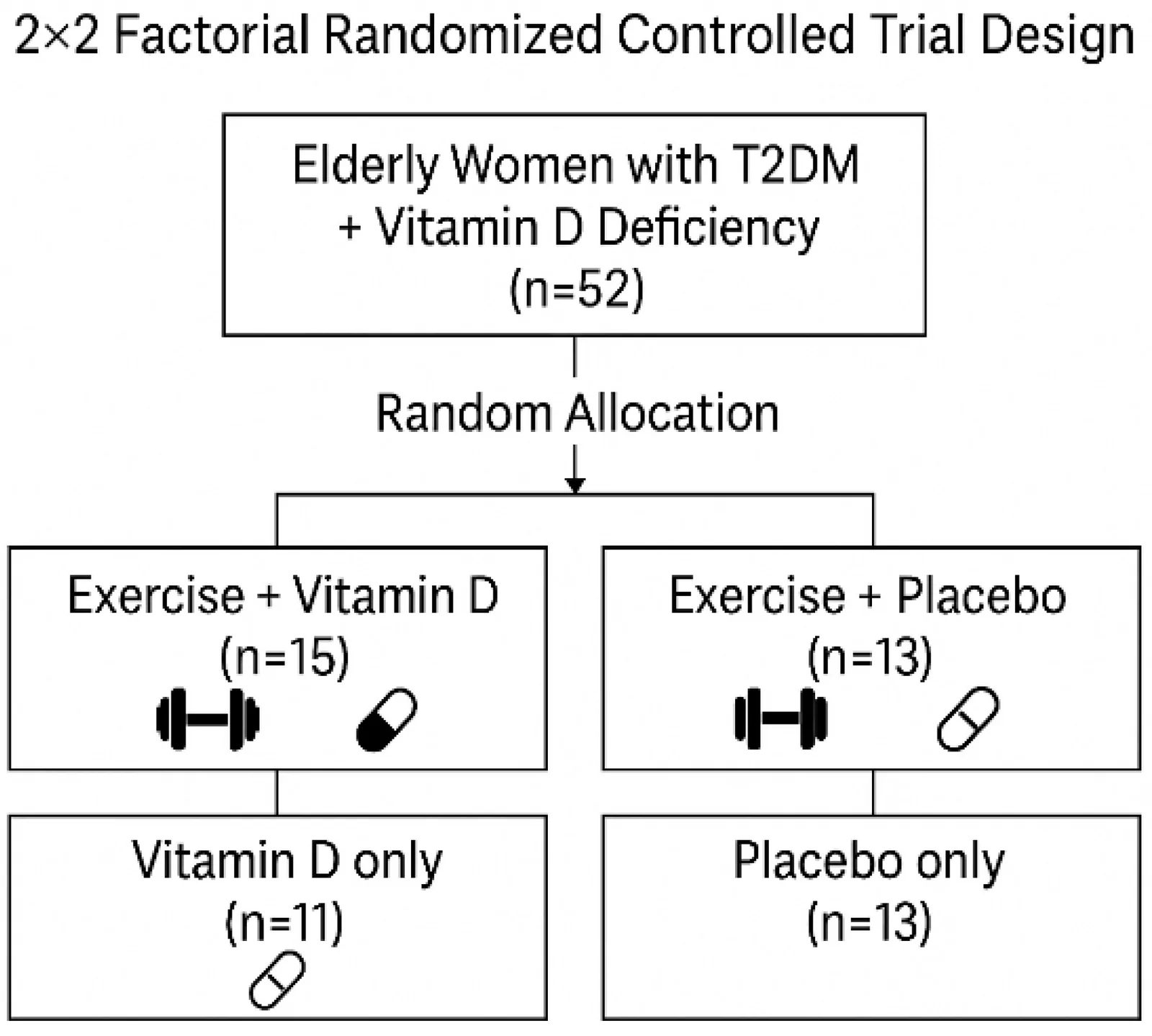
The 2 × 2 factorial randomized controlled trial design. Fifty-two elderly women with type 2 diabetes and vitamin D deficiency were allocated to Exercise + Vitamin D (*n* = 15), Exercise + Placebo (*n* = 13), Vitamin D only (*n* = 11), or Control (*n* = 13) to examine the independent and interactive effects of resistance exercise training and vitamin D supplementation.

The study was approved by the Institutional Review Board of Seoul National University Bundang Hospital on 9 March 2013 (No. B-1301/185-002) and conducted in accordance with the Declaration of Helsinki. All participants provided written informed consent. Participants received no direct monetary remuneration for their participation; however, all clinical health assessments, biochemical analyses, and the 12-week supervised exercise program were provided free of charge. Participant flow is shown in Figure 1, and the factorial design is summarized in Figure 2.

### 2.3. Interventions

#### 2.3.1. Resistance Exercise Training

Exercise groups completed supervised training three times weekly for 12 weeks (36 sessions). Each 60-min session comprised a 10-min warm-up, a 40-min resistance circuit (8–10 exercises: leg press, leg extension, leg curl, calf raise, squat, chest press, seated row, shoulder press, crunches, and back extension), and a 10-min cool-down. To optimize safety, minimize muscular strain, and promote participant adaptation, the program followed a periodized progression: Weeks 1–4 served as an initial familiarization and adaptation phase (50–60% 1-RM, 2 sets × 12–15 reps), progressing to Weeks 5–8 (60–70% 1-RM, 2–3 sets × 10–12 reps), and Weeks 9–12 (70–80% 1-RM, 3 sets × 8–10 reps). All exercise sessions were directly supervised by certified exercise specialists, with rest intervals (1–2 min) adjusted based on real-time subjective fatigue (RPE). High feasibility was demonstrated as no exercise-related injuries or adverse hypoglycemic events occurred during the 12-week intervention. All exercise sessions were directly supervised by certified exercise specialists (e.g., certified Clinical Exercise Physiologists/Health Exercise Managers and Physical Education Specialists) who possessed clinical expertise in exercise prescription and safety management for elderly populations with metabolic disorders (1:5–7 ratio). Pre- and post-session blood glucose and blood pressure levels were routinely checked. No exercise-related injuries or severe adverse events (e.g., severe hypoglycemia) were reported. Per-protocol analysis required ≥80% adherence (minimum 29 sessions).

#### 2.3.2. Vitamin D Supplementation

Vitamin D groups received cholecalciferol 2000 IU/day for 12 weeks. Placebo groups received identical capsules containing microcrystalline cellulose. Participants took one capsule daily with meals. Compliance was assessed by pill counts at weeks 4, 8, and 12 (≥80% required). All participants maintained their usual diets and medications and received standardized nutrition education. Additionally, daily sun exposure and outdoor physical activity were monitored using standardized self-reported lifestyle logs at baseline and weeks 4, 8, and 12, with no significant differences observed among the groups.

### 2.4. Outcome Measures

Trained, blinded research personnel conducted all assessments at baseline (week 0) and post-intervention (week 12). Participants maintained habitual physical activity and dietary patterns for 48 h before assessment and fasted overnight (≥10 h) before blood collection.

#### 2.4.1. Primary Outcomes

Primary outcomes were glycemic control (fasting plasma glucose by the glucose oxidase method; a 75-g oral glucose tolerance test with venous sampling at 0, 30, 60, 90, and 120 min; glucose area under the curve by the trapezoidal rule; HbA1c by high-performance liquid chromatography; and HOMA-IR), vitamin D status (serum 25(OH)D by electrochemiluminescence immunoassay; Cobas system, Roche Diagnostics, Mannheim, Germany), and bone metabolism (calcitonin and parathyroid hormone (PTH) by electrochemiluminescence immunoassay; serum calcium and phosphorus by standard biochemical analysis).

#### 2.4.2. Secondary Outcomes

Secondary outcomes were anthropometry (height, weight, BMI, waist and hip circumference, and waist-to-hip ratio); lipid profile (total cholesterol, triglycerides, HDL-C, and LDL-C by enzymatic colorimetry); muscular strength (handgrip by Takei TKK 5401; Takei Scientific Instruments Co., Ltd., Niigata, Japan, 30-s arm curl with a 2-kg dumbbell, 30-s chair stand, and sit-and-reach); and postural balance (one-leg stand, functional reach, tandem walk, and Posturomed dynamic assessment; Haider Bioswing GmbH, Pullenreuth, Germany). Blood samples were collected between 07:00 and 09:00 after an overnight fast, immediately processed, and stored at −80 °C until batch analysis. All biochemical measures showed intra-assay and inter-assay coefficients of variation < 5% and <8%, respectively.

### 2.5. Statistical Analysis

Data were analyzed using IBM SPSS Statistics (version 26.0; IBM Corp., Armonk, NY, USA) with α = 0.05 (two-tailed). Descriptive statistics were expressed as mean ± SD for continuous variables and as frequencies for categorical variables. Baseline comparisons used one-way ANOVA (continuous) and chi-square tests (categorical). The primary analysis was a 2 × 2 factorial repeated-measures ANOVA examining main effects (exercise, vitamin D), interactions, and time effects. Significant interactions prompted simple-effects analysis with Tukey’s HSD post-hoc test. Within-group changes were assessed by paired *t*-tests with Cohen’s d (0.2 = small, 0.5 = medium, 0.8 = large). ANCOVA with baseline covariates addressed variables showing baseline differences (*p* < 0.05). Per-protocol analysis excluded participants with <80% adherence. Normality (Shapiro–Wilk), variance homogeneity (Levene), and sphericity (Mauchly with Greenhouse–Geisser correction) were assessed.

## 3. Results

### 3.1. Participant Characteristics and Baseline Comparisons

Of 68 women initially screened, 52 met the eligibility criteria and were randomized to Ex + VitD (*n* = 15), Ex + Placebo (*n* = 13), VitD (*n* = 11), and Control (*n* = 13). All participants completed the 12-week intervention with adherence exceeding 85% for both exercise sessions and supplement consumption. Baseline characteristics are presented in Table 1. Groups were comparable for age, height, weight, BMI, waist-to-hip ratio, and blood pressure (*p* > 0.05); however, baseline waist circumference differed significantly across groups (*p* = 0.002), with the Ex + VitD and Ex + Placebo groups showing higher values than the Control and VitD groups.

### 3.2. Glycemic Control and Metabolic Outcomes

Changes in glycemic control, muscular strength, and physical function following the 12-week intervention are presented in Table 2. No significant time × exercise × vitamin D interactions were observed for HbA1c, fasting glucose, insulin, or HOMA-IR (all *p* ≥ 0.257). However, a significant time × exercise effect was identified for HOMA-IR (F = 4.290, *p* = 0.044, η*p*^2^ = 0.082), with borderline time × exercise and time × vitamin D effects for insulin (F = 3.788, *p* = 0.057, η*p*^2^ = 0.073; F = 3.276, *p* = 0.077, η*p*^2^ = 0.064, respectively) and a borderline time × vitamin D effect for HOMA-IR (F = 3.711, *p* = 0.060, η*p*^2^ = 0.072). Within-group analyses showed that the Ex + VitD group exhibited the greatest glycemic improvement, with HbA1c decreasing from 6.61 ± 0.60% to 6.48 ± 0.58% (Δ = −0.13 ± 0.20), fasting glucose from 7.37 ± 1.15 to 6.58 ± 1.10 mmol/L (Δ = −0.79 ± 0.56), insulin from 5.48 ± 4.24 to 3.52 ± 2.14μU/mL (Δ = ±3.55), and HOMA-IR from 1.80 ± 1.41 to 1.03 ± 0.66 (Δ = −0.77 ± 1.14). In contrast, the Ex + Placebo group showed smaller reductions, whereas the VitD and Control groups demonstrated minimal change or deterioration. Tukey’s post hoc analysis further showed that the change in insulin and HOMA-IR differed significantly between the Ex + VitD and Control groups (*p* = 0.039 and *p* = 0.025, respectively).

### 3.3. Vitamin D Status and Bone Metabolism

A robust time × vitamin D effect was observed for serum 25(OH)D (F = 41.347, *p* < 0.001, η*p*^2^ = 0.463), whereas no significant time × exercise × vitamin D interaction was detected (F = 0.975, *p* = 0.328, η*p*^2^ = 0.020). Serum 25(OH)D increased markedly in both vitamin D-supplemented groups, from 11.91 ± 6.42 to 27.48 ± 6.33 ng/mL in the Ex + VitD group (Δ = 15.57 ± 9.06) and from 10.44 ± 5.97 to 27.33 ± 8.56 ng/mL in the VitD group (Δ = 16.89 ± 9.32), whereas the Ex + Placebo and Control groups showed only minor changes. For calcitonin, a significant time × exercise effect was found (F = 10.640, *p* = 0.002, η*p*^2^= 0.181), although the time × exercise × vitamin D interaction did not reach significance (F = 2.124, *p* = 0.151, η*p*^2^ = 0.042). The largest increase was observed in the Ex + VitD group, from 4.82 ± 0.77 to 7.39 ± 1.60 pg/mL (Δ = 2.57 ± 1.78), and this change was greater than that in the VitD and Control groups (Tukey *p* = 0.009 and *p* = 0.003, respectively). For PTH, a significant time × vitamin D effect was identified (F = 8.724, *p* = 0.005, η*p*^2^ = 0.154), with values decreasing from 17.83 ± 8.79 to 10.80± 6.07 pg/mL in the Ex + VitD group (Δ = −7.03 ± 5.25) and from 13.22 ± 9.46 to 8.66 ± 3.37 pg/mL in the VitD group (Δ = −4.56 ± 9.13), whereas the Control group showed an increase (Δ = 3.50 ± 5.39).

### 3.4. Lipid Profile

No significant time × exercise × vitamin D interactions were observed for total cholesterol, triglycerides, HDL-C, or LDL-C (all *p* ≥ 0.233). However, significant time × exercise effects were detected for total cholesterol (F = 14.901, *p* < 0.001, η*p*^2^ = 0.237), HDL-C (F = 50.727, *p* < 0.001, η*p*^2^ = 0.514), and LDL-C (F = 17.344, *p* < 0.001, η*p*^2^ = 0.265). In addition, total cholesterol showed a significant time × vitamin D effect (F = 5.053, *p* = 0.029, η*p*^2^ = 0.095). In the Ex + VitD group, total cholesterol decreased from 191.73 ± 28.32 to 167.80 ± 23.84 mg/dL (Δ = −23.93 ± 22.45), triglycerides from 146.13 ± 54.50 to 121.07 ± 54.78 mg/dL (Δ = −25.07 ± 40.10), LDL-C from 104.80 ± 19.49 to 94.60 ± 19.92 mg/dL (Δ = −10.20 ± 12.39), and HDL-C increased from 53.60 ± 9.63 to 58.13 ± 10.41 mg/dL (Δ = 4.53 ± 5.30). The Ex + Placebo group also showed favorable changes in total cholesterol (Δ = −12.54 ±18.61), HDL-C (Δ = 3.31 ± 3.04), and LDL-C (Δ = −8.54 ± 12.89), whereas the VitD and Control groups showed either minimal change or an adverse direction, particularly for HDL-C and LDL-C. Tukey’s post hoc analysis confirmed greater reductions in total cholesterol and LDL-C, and greater increases in HDL-C, in the exercise-containing groups relative to Control and/or VitD.

### 3.5. Muscular Strength and Physical Function

Significant time × exercise effects were observed for left handgrip strength (F = 12.830, *p* < 0.001, η*p*^2^ = 0.211), right handgrip strength (F = 10.997, *p* = 0.002, η*p*^2^ = 0.186), arm curl performance (F = 12.650, *p* < 0.001, η*p*^2^ = 0.209), chair stand performance (F = 32.242, *p* < 0.001, η*p*^2^ = 0.402), and sit-and-reach flexibility (F = 10.328, *p* = 0.002, η*p*^2^ = 0.177), with no significant time × exercise × vitamin D interactions for these variables (all *p* ≥ 0.384) (Table 2). In the Ex + VitD group, left and right handgrip strength increased from 21.24 ± 3.71 to 23.46 ± 4.12 kg (Δ = 2.22 ± 1.90) and from 22.73 ± 3.38 to 23.98 ± 3.53 kg (Δ = 1.25 ± 2.08), respectively. In the Ex + Placebo group, corresponding increases were 1.98 ± 1.97 and 2.11 ± 2.09 kg. Arm curl performance increased by 5.67 ± 3.94 repetitions in the Ex + VitD group and by 5.77 ± 5.34 repetitions in the Ex + Placebo group, while chair stand performance increased by 8.33 ± 4.06 and 10.00 ± 5.20 repetitions, respectively. Sit-and-reach flexibility improved by 2.27 ± 2.00 cm in the Ex + VitD group and by 2.15 ± 1.48 cm in the Ex + Placebo group, whereas changes in the non-exercise groups were minimal. Tukey’s post hoc comparisons showed significantly greater gains in handgrip, arm curl, and chair stand performance in the exercise groups than in Control and/or VitD.

### 3.6. Postural Balance

For postural balance, a significant time × exercise × vitamin D interaction was observed for functional reach (F = 5.428, *p* = 0.024, η*p*^2^ = 0.102), together with a significant time × exercise effect (F = 10.982, *p* = 0.002, η*p*^2^ = 0.186). Functional reach increased from 33.33 ± 4.22 to 35.80 ± 4.63 cm in the Ex + VitD group (Δ = 2.47 ± 4.03) and from 32.46 ± 3.12 to 36.92 ± 4.41 cm in the Ex + Placebo group (Δ = 4.46 ± 4.48), while the Control group showed a decline (Δ = −2.38 ± 5.30); Tukey’s post hoc test confirmed significant differences between Control and Ex + VitD (*p* = 0.025) and between Control and Ex + Placebo (*p* = 0.001). Left one-leg stand time also showed a significant time × exercise effect (F = 4.635, *p* = 0.036, η*p*^2^ = 0.088) and a borderline time × vitamin D effect (F = 4.038, *p* = 0.050, η*p*^2^ = 0.078), whereas right one-leg stand time showed no significant factorial effects. Tandem walk time decreased only in the Ex + VitD group, from 8.28 ± 2.34 to 7.75 ± 2.38 s (Δ = −0.53 ± 0.65), but neither the time × exercise effect (F = 0.935, *p* = 0.338, η*p*^2^ = 0.019) nor the time × exercise × vitamin D interaction (F = 0.018, *p* = 0.895, η*p*^2^ < 0.001) was significant.

## 4. Discussions

This randomized trial shows that 12 weeks of progressive resistance training combined with vitamin D supplementation was associated with favorable improvements in metabolic and functional outcomes in elderly Korean women with T2DM and vitamin D deficiency, with factorial analyses indicating exercise-related effects for most strength- and function-related variables and a significant exercise × vitamin D interaction for functional reach.

### 4.1. Combined Metabolic Effects

Within-group analyses showed that the Ex + VitD group achieved a statistically significant HbA1c reduction (−0.13%, *p* = 0.019), substantial fasting glucose improvement (−14.20 mg/dL), and enhanced insulin sensitivity (HOMA-IR: −0.77); however, factorial analyses did not show significant exercise × vitamin D interactions for these glycemic outcomes. Although the absolute reduction of 0.13% is relatively modest and may have limited independent clinical magnitude compared to pharmacological agents, it remains notable given the brief 12-week non-pharmacological duration and the relatively well-controlled baseline (6.61%). Rather than reflecting a dramatic glycemic shift, this slight reduction, when accompanied by marked improvements in fasting glucose and HOMA-IR, indicates favorable early-stage metabolic stabilization. These findings align with the meta-analysis of Lee et al. [5], which reported HbA1c reductions of about 0.32% across vitamin D trials, although here they were achieved through combined rather than single interventions. The fasting glucose reduction exceeds improvements from vitamin D alone [6] and approaches the 16-week resistance-exercise findings of Castaneda et al. (−1.1% HbA1c) [8].

These outcomes suggest that resistance exercise and vitamin D supplementation may influence complementary metabolic pathways; however, because the exercise × vitamin D interaction was not statistically significant for the glycemic variables, this mechanistic complementarity should be interpreted cautiously.

### 4.2. Calcitonin as a Novel Mediator

The pronounced calcitonin elevation in the Ex + VitD group (+2.57 pg/mL, d = 3.34) is one of the most notable findings of this study; however, factorial analysis supported a significant exercise effect rather than a statistically significant exercise × vitamin D interaction. Because the largest calcitonin increase was observed in the Ex + VitD group, mechanical loading together with vitamin D repletion may have contributed to this response, although the formal interaction term was not statistically significant.

Although calcitonin is traditionally characterized as regulating calcium homeostasis [20,21], emerging evidence suggests broader metabolic roles. Klausen et al. reported acute calcitonin elevation during endurance exercise [18]; the sustained elevation observed here may contribute to metabolic improvements through enhanced glucose utilization, lipid oxidation, and anti-inflammatory effects. The substantial PTH reduction (−7.03 pg/mL) is consistent with vitamin D physiology, as adequate 25(OH)D suppresses inappropriate PTH secretion.

### 4.3. Lipid, Strength, and Balance Improvements

Both exercise groups showed favorable lipid alterations, and factorial analyses confirmed significant time × exercise effects for total cholesterol, HDL-C, and LDL-C, with the Ex + VitD group showing the largest reduction in total cholesterol (−23.93 mg/dL) and triglycerides (−25.07 mg/dL).

Concurrent HDL-C increases (+4.53 and +3.31 mg/dL) confer cardioprotective benefit. Non-exercise groups showed HDL-C reductions, suggesting that sedentary behavior promotes adverse lipid changes. The superior Ex + VitD lipid improvements corroborate the findings of Hoseini et al. that combined interventions upregulate hepatic PPARγ more effectively than monotherapies [4]. Substantial strength gains occurred across all domains (d = 0.37–3.65), with chair stand improvements particularly marked (Ex + VitD + 8.33 reps; Ex + Placebo + 10.00 reps), reflecting enhanced lower-extremity power crucial for fall prevention and demonstrating that elderly diabetic women retain substantial neuromuscular plasticity. Balance improvements, particularly in functional reach distance, occurred predominantly in the exercise groups, and functional reach was the only outcome showing a significant time × exercise × vitamin D interaction (F = 5.428, *p* = 0.024), although the largest absolute improvement was observed in the Ex + Placebo group (+4.46 cm, d = 1.43). The Ex + VitD group uniquely showed reduced tandem walk time (−0.53 s, *p* = 0.007) in within-group analysis; however, no significant factorial effect was observed for tandem walk, and this finding should therefore be interpreted cautiously.

The non-significant one-leg stand improvements likely reflect assessment limitations, with many participants reaching the 60-s maximum at baseline.

### 4.4. Comparison with Previous Research and Study Limitations

Our findings contrast with those of Kim et al., who reported no glycemic improvement after circuit training combined with vitamin D [14]. This discrepancy stems from fundamental differences in both intervention programming and statistical modeling sensitivity. Methodologically, Kim et al. utilized a combined aerobic–resistance circuit protocol, which dispersed cardiovascular and metabolic demands, thereby diluting the peak mechanical strain required to robustly drive skeletal muscle GLUT4 translocation and intracellular insulin signaling. Conversely, our current protocol applied pure progressive resistance training with strictly monitored 1RM-based load progression (60–75% 1RM) to deliver the threshold mechanical stimulus needed to enhance insulin sensitivity. Furthermore, analytical refinements account for this divergence: whereas earlier evaluations relied on standard linear comparisons, our trial employed a 2 × 2 factorial repeated-measures ANOVA alongside baseline-adjusted ANCOVA. This statistical framework allowed the independent and combined effects of exercise and vitamin D supplementation to be examined more rigorously; however, significant exercise × vitamin D interaction effects were limited, with clear evidence observed primarily for functional reach. Our results align with Sun et al., who documented improved insulin resistance after combined endurance exercise and vitamin D in middle-aged adults with T2DM [13]. Consistency across exercise modalities suggests that adequate physical activity combined with vitamin D repletion drives the metabolic benefits.

Study strengths include the factorial design examining independent and interactive effects, comprehensive outcome assessment, novel calcitonin measurement, and supervised training ensuring adherence. Limitations include the 12-week duration, which may underestimate long-term benefits; the exclusive focus on elderly Korean women, which limits generalizability; the modest vitamin D dose (2000 IU/day); and the absence of detailed postural control analysis. Although daily total energy intake was quantitatively monitored using 3-day dietary logs (analyzed via CAN-Pro 4.0) and participants were instructed to maintain their habitual dietary patterns without non-prescribed supplements, detailed micronutrient breakdown was limited, which may leave potential residual confounding. Furthermore, although all participants were required to be on stable medication regimens for at least 1 month prior to enrollment and throughout the 12-week intervention to prevent dosage-induced confounding, detailed itemized tracking of specific baseline drug classes, exact dosages, and potential drug–drug interactions was not recorded, remaining an inherent study limitation. Finally, while our findings generally align with recent systematic reviews and meta-analyses demonstrating favorable synergistic effects of combined physical activity and vitamin D supplementation on glycemic control and neuromuscular function [22], direct quantitative comparisons with broad meta-analytic findings remain limited due to our specific cohort characteristics and 12-week intervention duration. Future multi-center trials with larger sample sizes will be valuable to further evaluate these subgroup-specific interaction mechanisms within updated meta-analytic frameworks.

### 4.5. Clinical Implications

Combined progressive resistance training (three times weekly) and vitamin D supplementation (2000 IU/day) represents a safe, cost-effective intervention associated with favorable improvements within 12 weeks, particularly in exercise-responsive functional outcomes.

Healthcare providers should routinely screen elderly diabetics for vitamin D deficiency and prescribe combined interventions over isolated treatments. The substantial strength and balance improvements suggest reduced fall incidence, although longer trials with fall endpoints are needed. The marked calcitonin elevation in the Ex + VitD group suggests a potentially novel mechanistic pathway warranting further investigation, although confirmation in larger trials is needed because the formal interaction effect was not statistically significant.

## 5. Conclusions

Twelve weeks of progressive resistance training combined with vitamin D supplementation produced enhanced combined improvements in cardiometabolic profiles (glycemic control, insulin sensitivity, and lipid metabolism) and functional capacities (muscular strength and postural balance) in elderly women with T2DM and vitamin D deficiency. The Ex + VitD group achieved clinically meaningful reductions in HbA1c, fasting glucose, and HOMA-IR that neither intervention produced independently, alongside a dramatic calcitonin elevation not observed with monotherapies. These findings indicate that combined interventions target complementary biological pathways to optimize metabolic and functional outcomes in this high-risk population. Calcitonin emerges as a potential novel mediator linking mechanical loading and vitamin D status to metabolic improvement and warrants mechanistic investigation. Providers managing elderly individuals with concurrent T2DM and vitamin D deficiency should prioritize combined resistance training and vitamin D supplementation to achieve superior glycemic control, enhance muscular strength and functional capacity, and potentially reduce fall risk.

## Figures and Tables

**Table 1:** Baseline characteristics of participants.

Variable	Ex + VitD (*n* = 15)	Ex + Placebo (*n* = 13)	VitD (*n* = 11)	Control (*n* = 13)	*p*
Demographics					
Age (years)	71.3 ± 4.2	70.8 ± 3.9	72.1 ± 4.5	71.5 ± 4.1	0.845
Height (cm)	154.2 ± 5.1	153.8 ± 4.9	155.1 ± 5.3	154.5 ± 5.0	0.921
Weight (kg)	62.4 ± 8.7	61.9 ± 8.3	59.8 ± 7.9	60.2 ± 8.1	0.765
BMI (kg/m^2^)	26.2 ± 3.1	26.1 ± 2.9	24.8 ± 2.7	25.2 ± 2.8	0.512
WC (cm)	88.7 ± 8.4 ^a^	87.9 ± 8.1 ^a^	82.3 ± 7.2 ^b^	81.9 ± 7.0 ^b^	0.002
WHR	0.91 ± 0.06	0.90 ± 0.05	0.89 ± 0.06	0.89 ± 0.05	0.678
Blood pressure					
SBP (mmHg)	128.4 ± 12.3	129.1 ± 11.9	126.7 ± 13.1	127.8 ± 12.5	0.912
DBP (mmHg)	78.3 ± 8.7	79.1 ± 8.4	77.2 ± 9.1	78.5 ± 8.8	0.867
Glycemic control					
FPG (mmol/L)	7.37 ± 1.21	7.29 ± 1.18	7.41 ± 1.25	7.33 ± 1.19	0.956
HbA1c (%)	6.61 ± 0.82	6.58 ± 0.79	6.64 ± 0.85	6.60 ± 0.81	0.989
Insulin (μU/mL)	5.48 ± 2.31	5.41 ± 2.27	5.52 ± 2.35	5.46 ± 2.29	0.997
HOMA-IR	1.80 ± 0.91	1.76 ± 0.88	1.83 ± 0.93	1.79 ± 0.90	0.991
Vitamin D & bone markers					
25(OH)D (ng/mL)	11.91 ± 4.23	15.14 ± 4.57	10.44 ± 3.89	14.94 ± 4.51	0.067
Calcitonin (pg/mL)	4.82 ± 1.67	4.75 ± 1.63	4.89 ± 1.71	4.79 ± 1.65	0.994
PTH (pg/mL)	42.18 ± 12.34	41.93 ± 12.15	42.45 ± 12.51	42.07 ± 12.28	0.999
Lipid profile					
TC (mg/dL)	208.47 ± 34.21	207.85 ± 33.89	209.18 ± 34.67	208.15 ± 34.03	0.998
TG (mg/dL)	152.93 ± 45.67	151.69 ± 45.12	153.82 ± 46.23	152.38 ± 45.45	0.996
HDL-C (mg/dL)	48.13 ± 8.92	47.85 ± 8.67	48.45 ± 9.21	48.07 ± 8.89	0.994
LDL-C (mg/dL)	129.73 ± 28.45	129.31 ± 28.12	130.27 ± 28.89	129.62 ± 28.34	0.999

Data are presented as mean ± SD. BMI, body mass index; WHR, waist-to-hip ratio; WC, waist circumference; SBP, systolic blood pressure; DBP, diastolic blood pressure; FPG, fasting plasma glucose; HbA1c, glycated hemoglobin; HOMA-IR, homeostatic model assessment of insulin resistance; 25(OH)D, 25-hydroxyvitamin D; PTH, parathyroid hormone; TC, total cholesterol; TG, triglycerides; HDL-C, high-density lipoprotein cholesterol; LDL-C, low-density lipoprotein cholesterol. Different superscript letters (^a^, ^b^) indicate significant between-group differences (*p* < 0.05, Tukey’s HSD post-hoc test).

**Table 2:** Changes in glycemic control, muscular strength, and physical function after the 12-week intervention.

Variable	Group	Baseline (M ± SD)	Post-Intervention (M ± SD)	Δ (M ± SD)	Within-Group *p*	Between-Group Δ	Ex F (*p*)	VitD F (*p*)	Ex × VitD F (*p*)
HbA1c (%)	Ex + VitD	6.61 ± 0.60	6.48 ± 0.58	−0.13 ± 0.20	0.019 *	NS	2.200 (0.145)	1.893 (0.175)	0.914 (0.344)
	Ex + Placebo	6.59 ± 0.58	6.50 ± 0.60	−0.09 ± 0.23	0.172	—	—	—	—
	VitD	6.64 ± 0.85	6.56 ± 0.90	−0.08 ± 0.48	0.587	—	—	—	—
	Control	6.60 ± 0.81	6.75 ± 0.70	0.15 ± 0.45	0.261	—	—	—	—
Fasting glucose (mmol/L)	Ex + VitD	7.37 ± 1.15	6.58 ± 1.10	−0.79 ± 0.56	<0.001 ***	NS	2.693 (0.107)	1.366 (0.248)	0.052 (0.820)
	Ex + Placebo	7.06 ± 0.87	6.80 ± 1.17	−0.25 ± 0.74	0.240	—	—	—	—
	VitD	8.06 ± 1.81	7.99 ± 2.18	−0.07 ± 2.07	0.912	—	—	—	—
	Control	7.14 ± 1.58	7.43 ± 2.19	0.29 ± 1.77	0.564	—	—	—	—
Insulin (μU/mL)	Ex + VitD	5.48 ± 4.24	3.52 ± 2.14	−1.96 ± 3.55	0.050 *	Ex + VitD vs. Control (0.039)	3.788 (0.057)	3.276 (0.077)	1.316 (0.257)
	Ex + Placebo	5.52 ± 3.04	4.83 ± 2.43	−0.69 ± 4.31	0.574	—	—	—	—
	VitD	5.40 ± 3.92	4.97 ± 2.99	−0.43 ± 4.71	0.769	—	—	—	—
	Control	4.69 ± 3.14	9.95 ± 13.20	5.26 ± 11.76	0.133	—	—	—	—
HOMA-IR	Ex + VitD	1.80 ± 1.41	1.03 ± 0.66	−0.77 ± 1.14	0.020 *	Ex + VitD vs. Control (0.025)	4.290 (0.044) *	3.711 (0.060)	1.147 (0.290)
	Ex + Placebo	1.72 ± 0.93	1.50 ± 0.81	−0.21 ± 1.36	0.582	—	—	—	—
	VitD	1.83 ± 1.23	1.71 ± 1.14	−0.12 ± 1.61	0.812	—	—	—	—
	Control	1.54 ± 1.11	3.38 ± 4.77	1.84 ± 4.05	0.127	—	—	—	—
Handgrip strength (kg)	Ex + VitD	21.24 ± 3.71	23.46 ± 4.12	2.22 ± 1.90	<0.001 ***	Ex + VitD vs. Control (0.044); Ex + VitD vs. VitD (0.038)	12.830 (<0.001) ***	0.011 (0.916)	0.133 (0.717)
	Ex + Placebo	20.90 ± 2.60	22.88 ± 2.98	1.98 ± 1.97	0.004 **	—	—	—	—
	VitD	21.10 ± 2.35	21.28 ± 1.93	0.18 ± 2.34	0.802	—	—	—	—
	Control	21.71 ± 4.39	22.02 ± 3.94	0.32 ± 1.01	0.283	—	—	—	—
Arm curl (repetitions)	Ex + VitD	22.60 ± 4.10	28.27 ± 3.58	5.67 ± 3.94	<0.001 ***	Ex + VitD vs. Control (0.013); Ex + Placebo vs. Control (0.015)	12.650 (<0.001) ***	0.602 (0.442)	0.732 (0.397)
	Ex + Placebo	22.46 ± 2.82	28.23 ± 3.59	5.77 ± 5.34	0.002 **	—	—	—	—
	VitD	22.00 ± 5.92	24.18 ± 3.63	2.18 ± 4.87	0.169	—	—	—	—
	Control	23.08 ± 3.71	23.15 ± 5.01	0.08 ± 4.37	0.950	—	—	—	—
Chair stand (repetitions)	Ex + VitD	18.87 ± 3.66	27.20 ± 3.95	8.33 ± 4.06	<0.001 ***	Ex + Placebo vs. Control (<0.001); Ex + VitD vs. Control (0.002); Ex + Placebo vs. VitD(0.001); Ex + VitD vs. VitD (0.008)	32.242 (<0.001) ***	0.213 (0.646)	0.771 (0.384)
	Ex + Placebo	20.00 ± 2.74	30.00 ± 4.67	10.00 ± 5.20	<0.001 ***	—	—	—	—
	VitD	18.18 ± 4.24	20.55 ± 3.56	2.36 ± 3.07	0.029 *	—	—	—	—
	Control	18.85 ± 5.37	20.69 ± 4.94	1.85 ± 5.03	0.210	—	—	—	—
Sit-and-reach (cm)	Ex + VitD	14.91 ± 5.53	17.17 ± 5.04	2.27 ± 2.00	0.001 **	NS	10.328 (0.002) **	0.062 (0.804)	0.008 (0.927)
	Ex + Placebo	15.09 ± 5.37	17.25 ± 5.12	2.15 ± 1.48	<0.001 ***	—	—	—	—
	VitD	13.99 ± 5.13	14.03 ± 3.77	0.04 ± 2.11	0.955	—	—	—	—
	Control	9.15 ± 4.34	8.94 ± 4.57	−0.21 ± 3.96	0.853	—	—	—	—
Functional reach (cm)	Ex + VitD	33.33 ± 4.22	35.80 ± 4.63	2.47 ± 4.03	0.033 *	Ex + Placebo vs. Control (0.001); Ex + VitD vs. Control (0.025)	10.982 (0.002) **	0.470 (0.496)	5.428 (0.024) *
	Ex + Placebo	32.46 ± 3.12	36.92 ± 4.41	4.46 ± 4.48	0.004 **	—	—	—	—
	VitD	30.73 ± 4.88	32.00 ± 5.92	1.27 ± 3.20	0.216	—	—	—	—
	Control	30.73 ± 7.13	28.35 ± 5.22	−2.38 ± 5.30	0.130	—	—	—	—
Tandem walk (s)	Ex + VitD	8.28 ± 2.34	7.75 ± 2.38	−0.53 ± 0.65	0.007 **	NS	0.935 (0.338)	0.302 (0.585)	0.018 (0.895)
	Ex + Placebo	8.40 ± 2.33	8.13 ± 2.05	−0.27 ± 1.83	0.602	—	—	—	—
	VitD	10.50 ± 2.99	10.49 ± 2.94	−0.01 ± 3.30	0.991	—	—	—	—
	Control	9.94 ± 2.24	10.36 ± 2.82	0.41 ± 2.65	0.584	—	—	—	—

* *p* < 0.05, ** *p* < 0.01, *** *p* < 0.001. Δ indicates post-intervention minus baseline change. Within-group changes were assessed using paired *t*-tests. Between-group differences in change scores were examined using Tukey’s HSD post hoc test. Factorial statistics are presented as the main effects of exercise and vitamin D supplementation and their interaction. HbA1c, glycated hemoglobin; HOMA-IR, homeostatic model assessment of insulin resistance.

## Data Availability

The data presented in this study are available on request from the corresponding author. The data are not publicly available due to privacy restrictions.
